# Plasma viraemia in HIV-positive pregnant women entering antenatal care in South Africa

**DOI:** 10.7448/IAS.18.1.20045

**Published:** 2015-07-06

**Authors:** Landon Myer, Tamsin K Phillips, Nei-Yuan Hsiao, Allison Zerbe, Gregory Petro, Linda-Gail Bekker, James A McIntyre, Elaine J Abrams

**Affiliations:** 1 Division of Epidemiology & Biostatistics, School of Public Health & Family Medicine, University of Cape Town, Cape Town, South Africa; 2 Division of Medical Virology, University of Cape Town, South Africa; 3 National Health Laboratory Services, Cape Town, South Africa; 4 ICAP, Mailman School of Public Health, Columbia University, New York, NY, USA; 5 Department of Obstetrics & Gynaecology, University of Cape Town, Cape Town, South Africa; 6 Desmond Tutu HIV Centre, Institute of Infectious Diseases & Molecular Medicine, University of Cape Town, Cape Town, South Africa; 7 Anova Health Institute, Johannesburg, South Africa; 8 College of Physicians & Surgeons, Columbia University, New York, NY, USA

**Keywords:** viral load, pregnancy, antiretroviral therapy, prevention of mother-to-child transmission, South Africa

## Abstract

**Introduction:**

Plasma HIV viral load (VL) is the principle determinant of mother-to-child HIV transmission (MTCT), yet there are few data on VL in populations of pregnant women in sub-Saharan Africa. We examined the distribution and determinants of VL in HIV-positive women seeking antenatal care (ANC) in Cape Town, South Africa.

**Methods:**

Consecutive HIV-positive pregnant women making their first antenatal clinic visit were recruited into a cross-sectional study of viraemia in pregnancy, including a brief questionnaire and specimens for VL testing and CD4 cell enumeration.

**Results & discussion:**

Overall 5551 pregnant women sought ANC during the study period, of whom 1839 (33%) were HIV positive and 1521 (85%) were included. Approximately two-thirds of HIV-positive women in the sample (*n*=947) were not on antiretrovirals at the time of the first ANC visit, and the remainder (38%, *n*=574) had initiated antiretroviral therapy (ART) prior to conception. For women not on ART, the median VL was 3.98 log_10_ copies/mL; in this group, the sensitivity of CD4 cell counts ≤350 cells/µL in detecting VL>10,000 copies/mL was 64% and this increased to 78% with a CD4 threshold of ≤500 cells/µL. Among women on ART, 78% had VL<50 copies/mL and 13% had VL >1000 copies/mL at the time of their ANC visit.

**Conclusions:**

VL >10,000 copies/mL was commonly observed in women not on ART with CD4 cell counts >350 cells/µL, suggesting that CD4 cell counts may not be adequately sensitive in identifying women at greatest risk of MTCT. A large proportion of women entering ANC initiated ART before conception, and in this group more than 10% had VL>1000 copies/mL despite ART use. VL monitoring during pregnancy may help to identify pregnancies that require additional clinical attention to minimize MTCT risk and improve maternal and child health outcomes.

## Introduction

Plasma HIV viral load (VL) is the principle determinant of HIV transmission from mother-to-child (MTCT) during gestation, intrapartum and postpartum through breastfeeding, and reducing maternal VL is the primary mechanism of action of most antiretroviral interventions to prevent mother-to-child transmission (PMTCT) of HIV [1]. Despite the central role of VL in MTCT and its prevention as well as the significant new initiatives to eliminate MTCT globally, there is surprisingly little known about VL in populations of HIV-positive pregnant women entering PMTCT services in sub-Saharan Africa where most MTCT occurs. Until recently, CD4+ cell counts were the measure of choice to identify pregnant women with advanced HIV disease in need of lifelong antiretroviral therapy (ART) and, in turn, there have been substantial efforts to expand access to CD4 enumeration for PMTCT services across Africa over the past decade; during this time, access to VL testing has been restricted by costs and laboratory access [2]. As a result, current insights into HIV viraemia in pregnancy in African populations are limited to highly selected groups of pregnant women participating in intervention trials [3, 4]. Little is known about the distribution and determinants of plasma VL in the general population of pregnant women, nor the correlations between VL and CD4 cell count, particularly in high-prevalence settings.

## Methods

Between 1 April 2013 and 30 June 2014, we conducted a cross-sectional study of all HIV-positive pregnant women making their first antenatal care (ANC) visit at a large public sector primary care facility in Cape Town, South Africa. Coverage of ANC is high (>95%) in this setting, and PMTCT services including ART are integrated into ANC services [5]. Consecutive women were identified as HIV positive through two rapid antibody tests, and all women 18 years and older making their first ANC visit during the current pregnancy were included; women who had initiated lifelong ART or prophylaxis at another facility during the current pregnancy and were then referred for ANC at the study site were excluded. After providing informed consent, women completed a short questionnaire including current ART use based on self-report with confirmation through review of medical records, and underwent ultrasound screening for estimation of gestational age. For women on ART, adherence was assessed through self-reported number of missed doses in the preceding 30 days. All women underwent phlebotomy for CD4 enumeration via flow cytometry (Beckman Coulter) and HIV VL testing (Abbott RealTime HIV-1), with tests conducted by the South African National Health Laboratory Services.

Data were analyzed using Stata Version 13.0 (Stata Corporation, College Station, TX, USA). All analyses were stratified by women's current ART use. Chi-squared and rank-sum tests were used to compare proportions and medians between groups, respectively. For women not on ART, VL values were made binary around 10,000 copies/mL, as this represents the threshold for significant increases in transmission risk [6]; for women using ART, viraemia was defined at >1000 copies/mL, following local definitions of virologic failure. Measures of diagnostic performance for CD4 cell counts in detecting women with different levels of viraemia were calculated as proportions with exact 95% confidence intervals (CIs). Logistic regression was used to identify the predictors of raised plasma viraemia in women on ART, with results presented as odds ratios (OR) with 95% CI. The parent study was reviewed and approved by the Human Research Ethics Committee of the University of Cape Town Faculty of Health Sciences as well as the Institutional Review Board of the Columbia University Medical Center.

## Results

During the study period, 5551 women sought ANC at the study facility, of whom 1839 (33%) were HIV positive and 1521 (85%) were included. Among the 318 women not included, the most common reasons for exclusion were: initiation of antiretrovirals during the current pregnancy before seeking ANC at the study facility (72% of exclusions), not pregnant (7%) or age <18 years (7%); refusals were minimal (3%).

Overall, 62% of HIV-positive women in the sample (*n*=947) were not on antiretrovirals at the time of the first ANC visit, and the remainder (38%, *n*=574) were receiving ART. For women not on ART and women on ART, the median CD4 cell counts were 378 and 393 cells/µL, and the median VL was 3.98 and 1.69 (the lower limit of detection) log_10_ copies/mL, respectively. The median gestation at the first antenatal visit was 19 weeks and was similar for both groups (Table 1). Women not using ART were younger, had lower gravidity, higher education and were less likely to have previous PMTCT exposure, compared to women on ART. Among ART users, the median duration of ART use was 32 months, and the most common regimens reported included tenofovir with either efavirenz or nevirapine. Four percent of women not on ART during the current pregnancy reported defaulting ART, having started and then stopped triple-drug therapy previously.

**Table 1:** Description of 1521 consecutive HIV-positive pregnant women according to antiretroviral therapy use at the start of antenatal care

	Women not using ART (*n*=947)	Women using ART (*n*=574)	*p*
Median age (IQR)	27 (24–32)	31 (28–34)	<0.001
<24 years	255 (27)	51 (9)	<0.001
25–29 years	340 (36)	166 (29)	
30–34 years	241 (26)	215 (38)	
>35 years	108 (11)	140 (25)	
Median gravidity (IQR)	2 (2–3)	3 (2–3)	<0.001
Primagravida	182 (19)	61 (11)	<0.001
Gestation (weeks) (IQR)	19 (14–25)	19 (13–26)	0.910
≤14 weeks	249 (27)	158 (29)	0.168
>14 to ≤28 weeks	506 (56)	280 (51)	
>28 weeks	156 (17)	112 (20)	
Completed high school	284 (30)	129 (22)	0.001
Currently employed	348 (37)	216 (38)	0.551
HIV diagnosis in current pregnancy	511 (54)	0	
Married/cohabiting	400 (44)	274 (49)	0.067
Previous PMTCT use	259 (34)	241 (47)	<0.001
Previous NVP only	16 (2)	12 (2)	0.767
Previous NVP+AZT	244 (32)	229 (44)	<0.001
Characteristics of ART use			
Previous ART use, defaulted	38 (4)	0	
Currently using ART	0	574 (100)	
Median duration of ART use in months (IQR)	–	32 (17–60)	
Current ART regimen on self-report (*n*=311)	–		
TFV-3TC-EFV	–	165 (29)	
TFV-3TC-NVP	–	57 (10)	
Other NNRTI-based regimen	–	56 (10)	
PI-based regimen	–	31 (5)	
Other second-line regimen	–	2 (<1)	
Median CD4 cell count at start of antenatal care (cells/µL) (IQR)	378 (250–543)	393 (273–531)	
≤200	150 (16)	64 (11)	
201–350	263 (28)	171 (30)	
351–500	239 (26)	157 (28)	
>500	274 (30)	171 (30)	
Median HIV viral load at start of antenatal care (log_10_ copies/mL)	3.98	1.69	
<50 copies/mL	35 (4)	447 (78)	
50–1000 copies/mL	115 (12)	50 (9)	
1001–10,000 copies/mL	334 (35)	36 (6)	
10,000–100,000 copies/mL	362 (38)	30 (5)	
>100,000 copies/mL	98 (10)	11 (2)	

**Figure 1:**
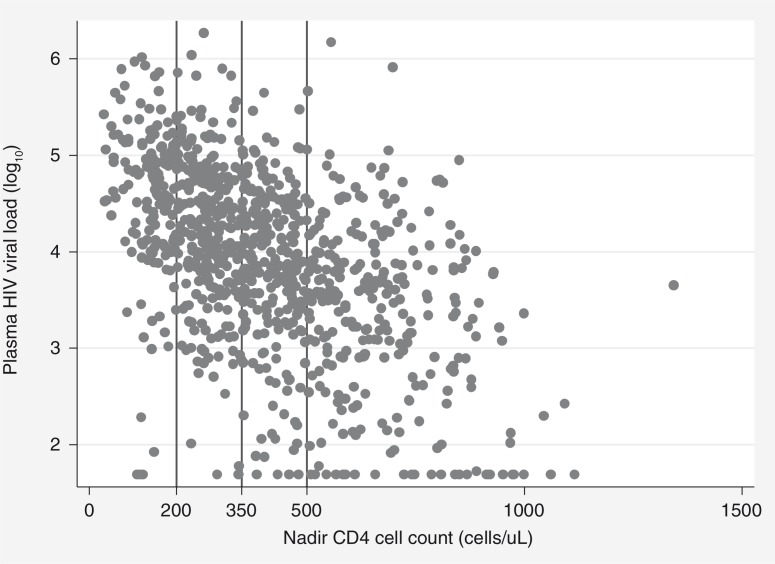
Scatterplot of plasma HIV viral load versus nadir CD4 cell count, among HIV-positive pregnant women not using antiretrovirals. Vertical lines denote CD4 thresholds of 200, 350 and 500 cells/µL.

Figure 1 shows the distribution of CD4 and VL measures for women entering ANC not using antiretrovirals. VL of <50, 50–1000, 1001–10,000, 10,001–100,000 and >100,000 copies/mL were observed in 4, 12, 35, 38 and 10% of women not on ART, respectively. The sensitivity of CD4 cell counts ≤350 cells/µL in detecting VL>10,000 copies/mL was 64% (36% of women with VL>10,000 copies/mL had CD4>350 cells/µL; 95% CI: 32%–41%) and this increased to 78% with a CD4 threshold of ≤500 cells/µL. In the group of women with VL>10,000 copies/mL not on ART, younger age was associated with higher CD4 cell counts and a lower sensitivity of CD4 cell counts in detecting VL>10,000 copies/mL (*p=*0.002), but no other factors appeared to modify the association between CD4 cell count and VL (not shown).

**Figure 2:**
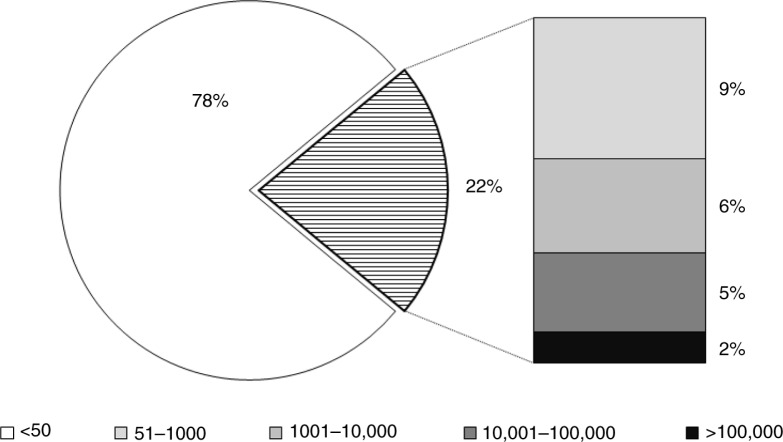
Distribution of plasma HIV viral loads in HIV-positive women conceiving on ART making their first antenatal clinic visit in Cape Town, South Africa. All values are in copies/mL.

Among women on ART, 22, 13 and 7% had VL >50, >1000 and >10,000 copies/mL, respectively (Figure 2). In simple logistic regression models restricted to women on ART at the time of the first ANC, raised VL>1000 copies/mL was associated with reporting two or more missed ART doses in the preceding 30 days (OR, 2.80; 95% CI, 1.54–5.07); in same model, lower VL was associated with being married or cohabiting (OR, 0.55; 95% CI, 0.33–0.92) and older age (OR for a one-year increase in age, 0.95; 95% CI, 0.91–1.00). In a multivariable model including age, marital status, years of ART use and previous PMTCT exposure, reporting two or more missed doses remained strongly associated with raised VL (adjusted OR, 2.39; 95% CI, 1.21–4.71).

## Discussion

To our knowledge, this is the largest study of VL in an unselected population of HIV-positive women in sub-Saharan Africa, and the data provide new insights into HIV viraemia in pregnant women in a high-prevalence setting. The key findings are that among ART-naïve women, CD4 cell counts fail to identify a large proportion of women with high VL, that there are a high proportion of women entering ANC already on ART, and in this group, a substantial proportion have detectable viraemia.

The distribution of VL in women who were not currently using ART, with 48% of women having VL>10,000 copies/mL, is somewhat lower than what has been described for non-pregnant adult populations from South Africa and Uganda [7, 8]. Given that there is no evidence that pregnancy alters HIV viraemia in women not on ART [9], this finding may be in keeping with the observation that fecundity requires that HIV-positive women be relatively healthy, and thus populations of pregnant women will be at earlier stages of HIV disease, on average, than non-pregnant women [10]. In addition, this VL distribution may help to explain the success of single-drug ARV regimens in reducing MTCT. We also found that roughly one-third of women with VL>10,000 had CD4 cell counts above 350 cells/µL (36%), and one-fifth (22%) had CD4 cell counts above 500 cells/µL. While we do not have data on timing of HIV infection, it is plausible that these discordant parameters may be more common in recently infected women. Using CD4-based eligibility criteria, these women would not be eligible for triple-drug antiretroviral regimens under various national and international guidelines. South Africa and other countries have recently shifted to policies of universal ART in pregnancy regardless of CD4 cell count [11], and these data suggest that such approaches may be required to reach all pregnant women with elevated VL at risk of vertical HIV transmission. Similarly, efforts to use ART to prevent secondary HIV transmission may be limited if CD4 count thresholds of 350 or 500 cells/µL are used to determine eligibility.

In this clinical setting, a large proportion of HIV-positive pregnant women entering PMTCT programmes have already initiated ART prior to conception. This is primarily a reflection of the relatively high levels of ART coverage locally, and while the proportion of women entering PMTCT programmes already on ART will vary across populations, it is likely to increase over time across the region as a growing number of women of reproductive age initiate therapy [12]. Historically, the interventions provided by PMTCT services in settings of high HIV prevalence have focused on ART-naïve women. However, these data suggest that in the future PMTCT services will require adaptation to focus on women conceiving after ART initiation. Here, we found that 22% of women already on ART had detectable viraemia; the causes of this viraemia require further attention, as these cross-sectional data are unable to distinguish transient “blips” from chronic viraemia due to non-adherence and/or viral resistance. Our finding that non-adherence was the strongest predictor of viraemia suggests ongoing adherence concerns in women of reproductive age and the need to strengthen adherence support as part of pregnancy planning services for women on ART.

The results should be interpreted in light of several limitations. Given the cross-sectional nature of this analysis, these data do not speak to VL changes over time, and we are unable to discern temporal associations involving VL and CD4 cell counts. The data come from a single community in urban South Africa with relatively high levels of ART coverage in women of reproductive age, and as a result, the proportion of women entering ANC having conceived on ART may be higher than in other settings. However, even in countries and communities with lower levels of ART coverage, the expansion of policies calling for universal initiation of lifelong ART in all HIV-positive pregnant women [11] means that women conceiving on ART may eventually become the majority of HIV-positive women entering PMTCT services. In addition, our measures of previous PMTCT exposure for all women and current ART regimen for women already on ART were based on self-report, and associations involving those measures should be interpreted with caution.

These data point to the potentially valuable information that may be provided by routine VL testing in HIV-positive pregnant women. There has been a shift recently to promote VL monitoring for patients on ART in resource-limited settings [13], and these data suggest that pregnant women on ART may benefit in particular from routine VL testing at the start of ANC to identify viraemic women at increased risk of MTCT. However, it is important to note that a single VL test may be difficult to interpret towards clinical management, and additional investigations are required to understand the aetiology of raised VL in these women. Given the time-delimited nature of pregnant and vertical transmission risk, the optimal strategies for VL testing and subsequent management of viraemia during pregnancy in women on ART require further attention. Meanwhile, the potential role of VL testing for pregnant women not on ART remains unclear; with expanding access to VL testing across sub-Saharan Africa, further operational research is required to investigate whether VL testing upon entry into PMTCT services may improve outcomes for HIV-positive mothers and their children.

It remains unclear what interventions should be introduced into routine care across sub-Saharan Africa to address elevated VL in pregnancy, particularly late gestation. In Europe or North America, elevated VL in a pregnant woman on ART may prompt urgent intervention, including an alteration in antiretroviral regimen and/or specific obstetric procedures such as caesarean section [14]. Yet in most parts of sub-Saharan Africa, both choices of antiretroviral agents and access to caesarean section are sharply restricted. In turn, the value of VL monitoring to assist PMTCT programmes in sub-Saharan Africa may be shaped by the ability to intervene when VL is elevated; with increasing emphasis on MTCT elimination globally, there is a clear need for evidence-based strategies that help identify and then mitigate against raised VL in pregnancy.

## Conclusions

In summary, this study presents important new data on plasma HIV viraemia in pregnancy. For women not on ART, high VLs were observed in women with relatively high CD4 cell counts, suggesting that CD4 cell counts may not be ideal to identify women at the greatest risk of MTCT. A substantial proportion of women entering ANC have already initiated ART before conception, and in these women, VL testing during pregnancy may help to identify women who require additional clinical attention to minimize MTCT risk and improve maternal health outcomes.
